# Insights into elections: An ensemble bot detection coverage framework applied to the 2018 U.S. midterm elections

**DOI:** 10.1371/journal.pone.0244309

**Published:** 2021-01-06

**Authors:** Ross J. Schuchard, Andrew T. Crooks

**Affiliations:** 1 Department of Computational and Data Sciences, George Mason University, Fairfax, Virginia, United States of America; 2 Department of Geography, University at Buffalo, Buffalo, New York, United States of America; 3 RENEW Institute, University at Buffalo, Buffalo, New York, United States of America; Universitat Rovira i Virgili, SPAIN

## Abstract

The participation of automated software agents known as social bots within online social network (OSN) engagements continues to grow at an immense pace. Choruses of concern speculate as to the impact social bots have within online communications as evidence shows that an increasing number of individuals are turning to OSNs as a primary source for information. This automated interaction proliferation within OSNs has led to the emergence of social bot detection efforts to better understand the extent and behavior of social bots. While rapidly evolving and continually improving, current social bot detection efforts are quite varied in their design and performance characteristics. Therefore, social bot research efforts that rely upon only a single bot detection source will produce very limited results. Our study expands beyond the limitation of current social bot detection research by introducing an ensemble bot detection coverage framework that harnesses the power of multiple detection sources to detect a wider variety of bots within a given OSN corpus of Twitter data. To test this framework, we focused on identifying social bot activity within OSN interactions taking place on Twitter related to the 2018 U.S. Midterm Election by using three available bot detection sources. This approach clearly showed that minimal overlap existed between the bot accounts detected within the same tweet corpus. Our findings suggest that social bot research efforts must incorporate multiple detection sources to account for the variety of social bots operating in OSNs, while incorporating improved or new detection methods to keep pace with the constant evolution of bot complexity.

## Introduction

The 2016 U.S. presidential election broke traditional campaign communication norms, as legacy institutions such as mainstream media sources (e.g. print, television and radio) and political-party organizations ceded much power and influence to unmediated, Internet-based technological platforms (e.g. online social networks (OSNs), online political blogs) [1]. Prior to 2016, Gibson and Cantijoch [2] had noted that there was an increasing number of people engaging in political discourse in OSNs (e.g. Twitter, Facebook) and described such behavior as a new type of expressive political engagement. Since the 2016 U.S. election, OSNs have surpassed print newspapers as a primary news source and continue to gain traction in relation to television and radio sources [3]. While the rapid rise of OSN platforms has reduced the barrier for individuals to actively participate in political dialogue, the relatively unsupervised nature of OSNs increases susceptibility to misinformation campaigns, especially with respect to political and election dialogue [4–6].

Social bots—automated software agents designed to mimic or impersonate humans—are prevalent actors in OSN platforms and have proven to amplify misinformation by orders of magnitude [7]. While the original design or purpose of social bots is not always nefarious, their impact can directly lead to the intentional or unintentional spreading of false narratives [8]. The inability for humans to readily discern whether they are engaging in dialogue with a human is a newly intractable problem with unknown implications. The rapidly evolving social bot problem has led to the recent emergence of numerous research efforts dedicated to the development of novel bot detection algorithms [9–12]. Moving beyond detection algorithm development, introductory social bot analysis efforts have also started to appear which have examined the prevalence and activities of detected social bots within general Twitter and Facebook conversations [13–15]. Further social bot analysis works have focused on detected bots within Twitter conversations involving specific topic areas such as the Brexit referendum [16,17], vaccinations [18], stock market trading [19], conflict [20] and political elections [21–24].

The constantly evolving sophistication of social bots has proven challenging for even the most promising detection algorithms developed to date [25]. This relates to the ever-expanding range of potential bot characteristics and activity patterns which demands continual refinement to existing detection methods or the development of entirely new methods to account for the most sophisticated bots. In summarizing the array of different detection approaches, Jiang et al [26] cautioned that detection applications, while looking to maximize the detection of the most ‘suspicious’ behaviors, employ different definitions of suspicious behaviors. In effect, the design parameters of bot detection algorithms will return results to which the algorithms are trained, and, thus, different detection strategies should detect different types of social bots. Recent efforts have focused on the evolving nature of bots by introducing adversarial learning detection algorithms [27,28]. While such detection advances are quite promising, they serve no immediate role in assisting broad, multidisciplinary social bot analysis efforts, since they are not readily accessible to the larger research community. Therefore, most current social bot analysis research efforts rely primarily upon an open-source bot detection platform service such as Botometer [9,29] or DeBot [10], which, like most detection algorithms, currently only focus on Twitter due to its ease of data accessibility via its publicly available standard application programming interface (API).

As the results of the 2015 Defense Advanced Research Projects Agency (DARPA) Twitter Bot Challenge summarized, no single detection algorithm is able to account for the myriad of social bots operating in OSNs [30]. It is from this perspective that the following study expands current social bot analysis research by incorporating multiple social bot detection services to determine the prevalence and relative importance of social bots within an OSN conversation of tweets. Through the lens of the 2018 U.S. midterm elections, harvested tweets capturing the election conversation were analyzed for evidence of bots using three bot detection platform services: Botometer [29], DeBot [10] and Bot-hunter [11]. The resulting suspected bot evidence serves as the basis for an ensemble of applied social network analysis (SNA) methods to determine the relative structural importance of bots in the conversation. Finally, a comprehensive, ensemble bot detection coverage analysis evaluates the resulting overlap in performance among the employed bot detection services.

The results of this study show that bot and human accounts contributed temporally to our 43.5 million tweet election corpus at relatively similar cumulative rates. The multi-detection platform comparative analysis of intra-group and cross-group interactions shows that bots detected by DeBot and Bot-hunter persistently engaged humans at rates much higher than bots detected by Botometer. Furthermore, while bots accounted for less than 8% of all unique accounts in the election conversation retweet network, bots accounted for more than 20% of the top-100 and top-25 ranking out-degree centrality, thus suggesting persistent activity to engage with human accounts. Finally, the bot coverage overlap analysis shows that minimal overlap existed among the bots detected by the three bot detection platforms, with only eight total bot accounts detected by all.

The intra-group and cross-group analysis of the constructed retweet network shows that bots detected by DeBot and Bot-hunter persistently engaged humans at rates much higher (5.03% and 6.09%, respectively) than bots detected by Botometer (2.27%). In addition, the intra-group and cross-group interactions, when viewed from a consolidated bot account perspective, provide the first piece of evidence that minimal overall overlap existed between the set of bots detected by each detection platform. The centrality ranking results showed that bots, from an overall perspective, achieved large volumes of high centrality ranking positions despite their relatively small populations size. The classification of relative importance by social bot accounts was most noticeable with bots detected by DeBot in the out-degree rankings and with bots detected by Botometer in the eigenvector rankings. Analysis of the overlap of bots detected by the detection platforms showed that no overlap existed between the bots ranking in the top-50 centrality results. Moreover, the Jaccard similarity index showed little bot detection overlap from a pairwise perspective, while only eight bots out of a total of 254,492 unique bots in the overall tweet corpus were detected by all three detection platforms.

In the remainder of this paper, the **Background** section provides the necessary context for this study by introducing applicable previous works involving social bot detection and analysis. Next, the **Data and Methods** section details the specific data acquisition and processing, as well as the applied methods, used in this study. The **Results and Discussion** section presents the pertinent findings of the study, and the paper closes with the **Conclusion** section.

## Background

OSN research has emerged and evolved rapidly in concert with the global adoption of social media platforms throughout the past decade. While the limitations, biases and risks associated with using OSN data are widely discussed [31,32], there have been many positive insights gained from OSN research contributions. Such works include OSN-findings related to disaster event detection [33,34], suicide prevention and detection [35,36] and cyberbullying [37,38]. OSNs have even been described as transformational media in creating new avenues of political participation and dialogue [1,39], while also fostering strong patterns of rumor propagation driven by echo chambers [40]. In a 61-million person Facebook experiment during the 2010 U.S. congressional elections, Bond et al. [41] showed how social human ties were instrumental in spreading both online and offline political behavior. Vaccari et al. [42] identified that lower-threshold political engagement activities in OSNs, such as posting political views, were strongly associated with higher-threshold activities such as campaigning for particular parties/candidates and attending offline political events. In a survey of active political Twitter users, Bode and Dalrymple [43] discovered that a primary reason for engaging in political discourse on Twitter was due to a general lack of trust in mainstream media sources.

The increasing use of OSNs for political communication dialogue has led to the rightful criticism of the transparency and validity not only behind how social media platforms operationally promote certain narratives, but also of how the platforms verify accounts as human actors or social bots [44]. Not surprisingly, given the propensity for polarization and the observed emergence of echo chambers within political conversations in OSNs [45], social bot campaigns view the manipulation of political dialogue as a natural attack vector. With the emergent role of OSNs in the 2016 U.S. presidential election, as previously mentioned, recent social bot analysis efforts have expanded their focus greatly into political OSN conversations. These works include the examination of detected bots within the 2016 U.S. presidential election [4,21,22], the UK-EU Brexit referendum [16,17], the 2018 Italian general election [46], the 2017 Catalan referendum [47] and the 2019 Spanish general election [48] within Twitter conversations. These election-focused social bot analyses relied upon an assortment of bot detection platform algorithms, but they all used a single method to classify bots. Further, while these recent works produced promising results using a single bot detection method (e.g., Botometer in [17,21,22,48] or DeBot in [20]) and inspired the development of more robust detection algorithms, such as the vastly improved methods involving adversarial detection approaches [27,28], they ultimately do not support more robust analyses given the lack of accessibility to the underlying detection algorithms for other researchers. This study significantly expands this body of work by aggregating the classification results of three bot detection platforms (i.e., DeBot, Bot-hunter and Botometer) in an effort to provide a more holistic social bot analysis framework. The following introduces and highlights the three detection platform services employed in this study to classify bots within the 2018 U.S. midterm Twitter conversation. These particular detection platforms were chosen due to their open accessibility to researchers.

Botometer, a widely used open-source bot detection platform created by researchers at Indiana University, is based on a supervised Random Forest ensemble classification technique that evaluates more than 1,000 extracted features for each analyzed Twitter account [9,29]. Given the supervised nature of the underlying algorithm, Botometer requires and has updated its detection classification algorithm multiple times by retraining against new data [29,49]. Botometer ultimately provides a likelihood estimate score on a [0,1] scale that an account is a bot, with simple bots scoring (0.8–1.0) and more sophisticated (i.e. human-like) bots scoring (0.5–0.7) [29]. While popular, Botometer is limited by several significant factors, which have been thoroughly documented in previous works [47,50,51]. These limiting factors include an inability to retrospectively analyze historical tweets and to classify suspended/protected Twitter accounts, while its publicly available API does not support large-scale analyses given inherited Twitter API rate limits.

DeBot, an open-source bot detection platform developed by researchers at the University of New Mexico, adopts an unsupervised warped correlation method to detect and label as bots those Twitter accounts having more than 40 synchronous events in a given window of time [10]. This novel unsupervised implementation extends beyond just a traditional correlation analysis by incorporating the concept of time warping distance to identify correlative activities within a specific time sampling window. The DeBot binary classification scheme (i.e. bot or not) detects bots with high precision, but it does so at a cost of total recall due to the limited sample size of overall Twitter accounts it evaluates [52]. While limited in coverage and susceptible to the precision/recall tradeoff of bot detection highlighted by Morstatter et al. [53], historical DeBot results are easily accessible and have led to the identification of bot impact within social bot analyses [20,54].

Finally, Bot-hunter, a bot detection platform developed by researchers at Carnegie Mellon University, applies a supervised Random Forest classification method to previously extracted Twitter data in a multi-tiered fashion with successive tiers incurring higher computational costs [11]. This deliberate tiered approach overcomes the limitations observed with Botometer (i.e. scalability and the classification of suspended accounts) by allowing bot classification to occur locally and against historical tweets, as opposed to classification in coordination with the Twitter API. Further, the scale and reach of Bot-hunter allows for a more complete evaluation of Tweet corpus accounts, thus overcoming the recall tradeoff observed with the limited coverage provided by DeBot. In a similar fashion to Botometer, Bot-hunter returns a bot classification score for each Twitter account of interest on a normalized scale between 0 and 1. While Bot-hunter is not currently accessible via a public API, it was made available to this study by the Carnegie Mellon research team upon request.

## Data and methods

**Fig 1:**
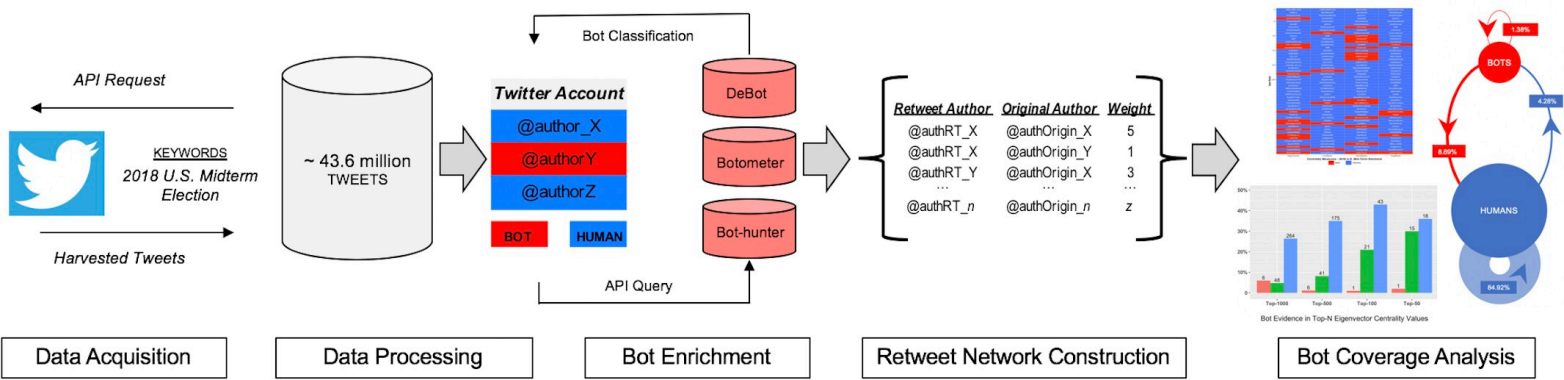
Social bot analysis framework employing multiple bot detection platforms. The framework enables the application of ensemble analysis methods to determine the prevalence and relative importance of social bots within Twitter conversations discussing the 2018 U.S. midterm elections.

This study breaks new ground in its use of multiple bot detection platforms to identify and analyze the presence of social bots within the 2018 U.S. midterm election OSN conversation. The following section details the study’s overall methodological framework as depicted in Fig 1. First, **Twitter Data** provides the essential background describing the capture, storage and processing stages required to develop the election midterm tweet corpus. **Bot Detection** details the steps taken to label the accounts within the election corpus with the three chosen bot detection platforms. **Retweet Network** construction explains the process to derive a network structure out of the original election conversation corpus. The section concludes with **Bot Analysis**, which introduces the applied analysis methods used in the remainder of the paper.

### Twitter data

The 2018 U.S. midterm elections provided a new opportunity to build upon previous social bot analyses dedicated to examining the role of bots within OSN election conversations. Given the specific limitations of bot detection platforms as described in the **Background** section, it was essential to properly prepare a collection plan well in advance of the planned 30-day collection window leading up to election day (November 6, 2018). As Zhang et al. [55] asserts, keyword selection in social media studies can induce varying levels of selection bias. To mitigate this risk, this study chose a comprehensive panel of keywords shown in Table 1 to capture the 2018 midterm election corpus. This panel included generic keywords associated with the election (e.g. Election2018, midterms2018) as well as keywords referencing campaign phrases and high-profile races in order to account for both major U.S. political parties.

**Table 1:** Election-related keywords submitted to capture relevant tweets associated with the 2018 U.S. midterm elections via the Twitter API.

Generic Election		Campaign Phrases		Key Races
Election2018	midterms	BlueWave	RedWave	@ScottWalker
midterms2018	2018midterms	FlipTheSenate	maga	@WISuptTonyEvers
democrat	republican	FlipTheHouse	kag	@tedcruz
DNC	RNC	VoteThemOut	buildthewall	@BetoORourke
DNC2018	RNC2018	HandsOffOurCare	takeitback	@SenatorHeitkamp
@TheDemocrats	@GOP			@KevinCramer
@SenateDems	@SenateGOP			@FLGovScott
@HouseDemocrats	@HouseGOP			@ SenBillNelson

The tweet collection process consisted of submitting the keyword panel to the publicly available Twitter standard streaming API for four weeks prior to the election day (October 10 through November 6, 2018). The overall tweet collection process yielded a consolidated corpus consisting in excess of 43.5 million tweets produced by approximately 3.2 million unique accounts. Retweets accounted for approximately 83.2% of the tweet corpus with more than 36.2 million retweets produced by more than 2.3 million unique accounts. Due to the large volume of harvested tweets and the subsequent data processing requirements as detailed in the remainder of this section, all immediate data processing and storage took place in a scalable 16vCPU and 64GB RAM Amazon Web Services (AWS) m5a.4xlarge instance.

### Bot detection

To detect and label social bots in the collected election conversation corpus, this study relied upon three bot detection platforms: Botometer, DeBot and Bot-hunter. While the **Background** section provided a general overview of these platforms and their underlying detection algorithms, the remainder of this subsection presents the technical details explaining how this study used each detection platform to detect and label bots within the election conversation corpus of tweets. First, a technical explanation describes the processing and environmental considerations associated with each platform. Next, given the scoring scales of Botometer and Bot-hunter, a scoring analysis explains the chosen cutoff threshold for labeling accounts as bots. Finally, an aggregate and specific detection platform perspective presents the bot detection results.

Currently, both DeBot and Botometer provide researchers open-source access to their hosted detection platforms via an API. However, due to individual API limitations, these two platforms required special access considerations to scale to the size of this study’s tweet corpus. Upon request, the DeBot development team provided access to the entire DeBot archival repository. The resulting detection processing simply consisted of matching unique tweet account information from the election conversation corpus to discovered bot profiles in the DeBot repository. The Botometer API provides both an open-access free tier with a rate limit of 17,280 requests per day and a ‘professional’ paid tier, which aligns to the publicly available Twitter standard API rate limits, with a rate limit of 43,200 requests per day. Due to the size of the election corpus and Botometer’s reliance on evaluating associated tweet data directly via the Twitter API, this study required three Botometer professional paid tier licenses in order to process the entire corpus volume in a timely manner. The faster execution tried to help mitigate Botometer’s inability to process suspended or deleted accounts by evaluating accounts prior to their potential removal by Twitter. As noted above, Bot-hunter does not currently provide a publicly available API, so the Bot-hunter team provided access to their platform upon request to process the raw tweets comprising the election conversation corpus.

**Fig 2:**
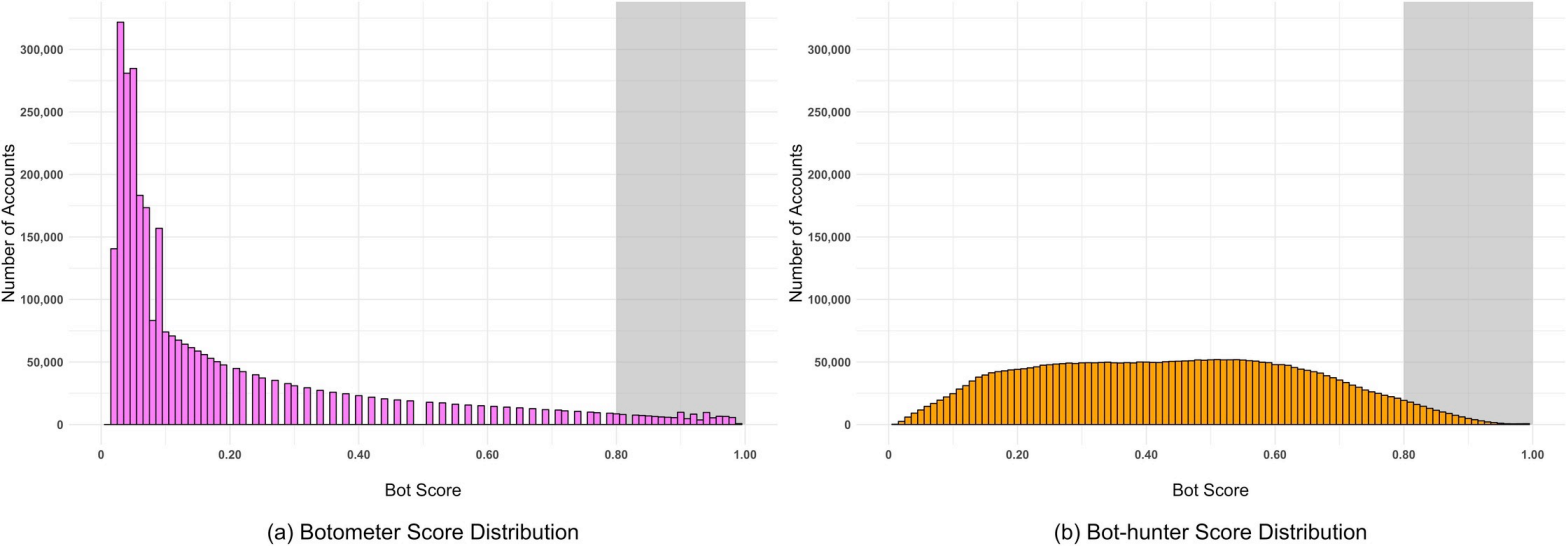
Resulting distribution of scores for Twitter accounts present within the 2018 U.S. midterm election tweet corpus using the (a) Botometer (pink) and the (b) Bot-hunter (orange) bot detection platforms.

Both Botometer and Bot-hunter return a classification score for each of the accounts they evaluate that falls within a [0,1] distribution, with a higher valuation constituting a greater likelihood that an account is a bot. DeBot, as previously mentioned, provides a simple binary classification for an account. Many studies using Botometer have historically used a 0.50 score threshold to classify bots [15,22,56]. While a clear binary cutoff threshold is a challenging decision to make, platforms like Botometer are providing the necessary transparency for researchers to make an informed decision [49]. This study used a highly conservative cutoff threshold of 0.80 to 1.00 to label accounts as detected bots, in a similar categorization paradigm of ‘most likely’ bots put forth by Broniatowski et al. [57]. This decision reflected a desire to determine the coverage overlap of the most certain bot accounts between different bot detection platforms. Fig 2 depicts the distribution of classification scores for both Botometer (Fig 2A) and Bot-hunter (Fig 2B), with the shaded gray areas highlighting the 0.80 to 1.00 score range.

Table 2 provides a summary of the bot detection classification volume results across all three bot detection platforms, as well as an aggregate classification volume. The aggregate classification method labels an account as a bot if at least one of the bot detection results declares that account to be a bot. In total, the aggregate bot classification process labeled 254,492 unique accounts, or 7.95% of all accounts, as bots that were responsible for contributing more than 5.7 million tweets (13.23% of all tweets) in the election corpus. From the specific detection platform perspective, Bot-hunter led all platforms by labeling 6.26% of all accounts as bots, followed by Botometer and DeBot with labeling rates of 3.80% and 0.64%, respectively. In terms of retweets, aggregate and specific platform bot labeling occurred at approximately that same rates; however, Botometer-labeled bot accounts retweeted at far lower rates in comparison to their regular tweet contribution rates.

**Table 2:** Twitter corpus volume and contributor populations from the 2018 U.S. midterm election OSN conversation with associated bot detection platform classification results.

Corpus	Detection Platform	Volume	% of Total	Contributors	% of Total
Tweets		43,565,164		3,201,996	
*Humans*		*37*,*800*,*157*	*86*.*77%*	*2*,*947*,*504*	*92*.*05%*
*Bots*		*5*,*765*,*007*	*13*.*23%*	*254*,*492*	*7*.*95%*
	DeBot	2,201,858	5.05%	20,605	0.64%
	Botometer	4,239,870	9.73%	121,780	3.80%
	Bot-hunter	2,729,354	6.26%	130,553	4.08%
Retweets		36,264,206		2,588,956	
*Humans*		*31*,*242*,*038*	*86*.*15%*	*2*,*388*,*447*	*92*.*26%*
*Bots*		*5*,*022*,*168*	*13*.*85%*	*200*,*509*	*7*.*74%*
	DeBot	1,991,654	5.49%	19,466	0.75%
	Botometer	920,675	2.54%	87,590	3.38%
	Bot-hunter	2,337,760	6.45%	107,861	4.17%

### Retweet network construction

A retweet serves as an observable interaction within a Twitter conversation that has been shown to promote trust [58] and increase engagement between users [59]. This study focused on retweets as the primary interaction of interest between accounts within the election conversation corpus. By extracting the directional nature of a retweet between two accounts, a logical node-edge paradigm emerges that can lead to the construction of an overall retweet network. For example, an initial retweet between two accounts receives a directional edge weight of ‘1’ and the edge weight increases by ‘1’ for each subsequent directional retweet between the same two accounts. Overall, the election corpus produced a retweet network, which served as the inherent graph object to enable the application of the SNA techniques described in the subsequent Bot Analysis Methods section, consisting of 3,388,805 nodes and 27,607,691 edges. The total network exhibited an average degree of 8.147. While not a fully connected network, the big component of the network consisted of 3,196,932 nodes, which accounted for 94.3% of the total network nodes.

### Bot analysis methods

The following subsections introduce the specific analytic methods used to determine the prevalence, characteristics and relative importance of detected bots within the 2018 U.S. midterm election conversation corpus. Each method accounted for bots from an aggregate labeling perspective, as well as for each bot detection platform. The description for each associated analysis method includes the specific data requirement and any theoretical references necessary to enable the most interpretive context of results presented in the **Results and Discussion** section.

#### Contribution rate analysis

Comparatively analyzing the temporal contribution patterns of bots and humans over time provided an opportunity to directly observe potential behavioral differences between the two sub-populations. Furthermore, this comparative context applied to differentiating the contribution patterns of bots detected by the various detection platforms used in this study. To accomplish this analysis, the entire election tweet corpus was divided into aggregate bot and human sub-populations. The resulting bot and human tweet contribution activities were then temporally indexed, resulting in a daily contribution rate. This same process was extended to the individual detection platform bot classification results. The **Results and Discussion** section presents the consolidated findings of the cumulative contribution rate analysis.

#### Intra-group and cross-group participation analysis

The constructed retweet network of the election conversation corpus enabled the observation of a multitude of communication interactions between bot and human accounts. These specific interactions can be reduced to intra-group (i.e. bots retweeting bots or humans retweeting humans) or cross-group (i.e. bots retweeting humans or humans retweeting bots) communication. To quantify the intra-group and cross-group communication volumes, applicable edgelists were created for each potential interaction. This included edgelists capturing the aggregate bot and human population interactions, as well as bot and human populations resulting from the individual bot detection platform results. These edgelists served as the foundational data source used to construct the visualization and associated results narrative presented in the **Results and Discussion** section.

#### Centrality ranking and bot coverage analysis

Beyond the examination of prevalence and behavioral characteristics, it is reasonable to attempt to ascertain whether social bots can be construed as ‘important’ actors within an OSN conversation. SNA centrality measures provide an efficient means to make such an assessment. Centrality measures can imply relative node importance based on a given node’s structural position in relation to other nodes within a network [60]. Social media research includes numerous applications of centrality analysis to determine the relative influence of contributing users in tweet networks [61]. Following the aforementioned node-edge characterization of retweets between accounts, this study applied the following four centrality measures that are efficiently scalable to the election corpus retweet network: eigenvector, in-degree, out-degree and PageRank.

Each of the applied centrality measures is a proxy for a specific form of relative importance within a retweet network. In-degree and out-degree centrality serve as a basis of popularity, given the cumulative direct inbound and/or outbound edges, or communication interactions, associated with each user account. Eigenvector centrality, which can be viewed as global measure of influence, is a more complex variant of degree centrality derived from the weighted sum of a given node’s complete set of direct and indirect edge connections. Finally, PageRank, is an extension of eigenvector centrality that weights a degree valuation higher for nodes that initiate edges with nodes that have the highest relative importance values [62]. Therefore, user accounts with the highest PageRank valuations in a retweet network are the recipients of more retweets from the most popular user accounts. Ranking the centrality results then allowed for the identification of the specific bots with relative structural importance, while also providing an opportunity to observe any redundant coverage between the detection platforms. In addition, the proposed method of ranking centrality results maintains the integrity of the ordinal ranking results of measures such as PageRank, which cannot produce an average global interpretation as attempted in other studies [47]. The **Centrality Ranking and Bot Coverage** subsection within the **Results and Discussion** section presents these results.

## Results and discussion

The following section presents the detailed results of the applied analysis methods described in the previous **Data and Methods** section. Based on the bot detection results from three bot detection platforms, the **Cumulative Bot Contribution Rates** subsection facilitated the comparative analysis of bot and human temporal contributions to the overall 2018 U.S. midterm election OSN conversation. The **Intra-Group and Cross-Group Comparison** subsection details the interaction patterns between human and bot accounts. This section concludes with the **Centrality Ranking and Bot Coverage** subsection identifying social bots within the centrality analysis ranking results, while also presenting a bot coverage assessment based on the results of the detection platforms used in this study.

### Cumulative bot contribution rates

**Fig 3:**
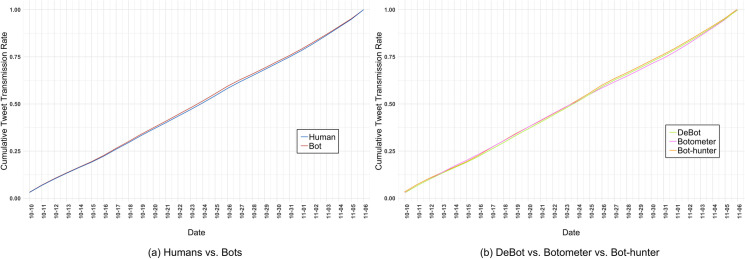
Cumulative tweet contribution rates for the 2018 U.S. midterm OSN conversation (October 10 –November 6, 2018) from the (a) human (blue) / bot (red) and (b) DeBot (green) / Botometer (pink) / Bot-hunter (orange) account classification perspectives.

Fig 3 presents the cumulative contribution rates of bot and human accounts to the 2018 U.S. midterm election OSN conversation. The results shown in Fig 3A directly compare human and bot contributions rates, with an account being classified as a bot if any of the study’s three detection platforms positively detected it as such. Visually, the contribution patterns of both human and bot accounts are quite consistent throughout the four weeks, although bot accounts slightly outpace the daily cumulative contributions of human accounts for the entire period. Fig 3B directly compares the cumulative contribution rates of bot accounts according to the bot detection classification results for each of the detection platforms. The results initially show similar cumulative contribution rates by bots from each detection platform, but bot accounts detected by DeBot and Bot-hunter outpace Botometer-detected bots from September 25^th^ through the November 6^th^ election day. It is surprising to see the relatively consistent contribution rates across both analysis scenarios, which could suggest that the Twitter election conversation elicited stable attention from both bot and human account contributors. While requiring further analysis, the observed cumulative contribution divergence by Botometer bots from DeBot and Bot-hunter bots midway through the conversation collection period could potentially suggest that bots detected by Botometer shift their interest over time to conversational topics beyond the election discussion.

### Intra-group and cross-group comparison

**Fig 4:**
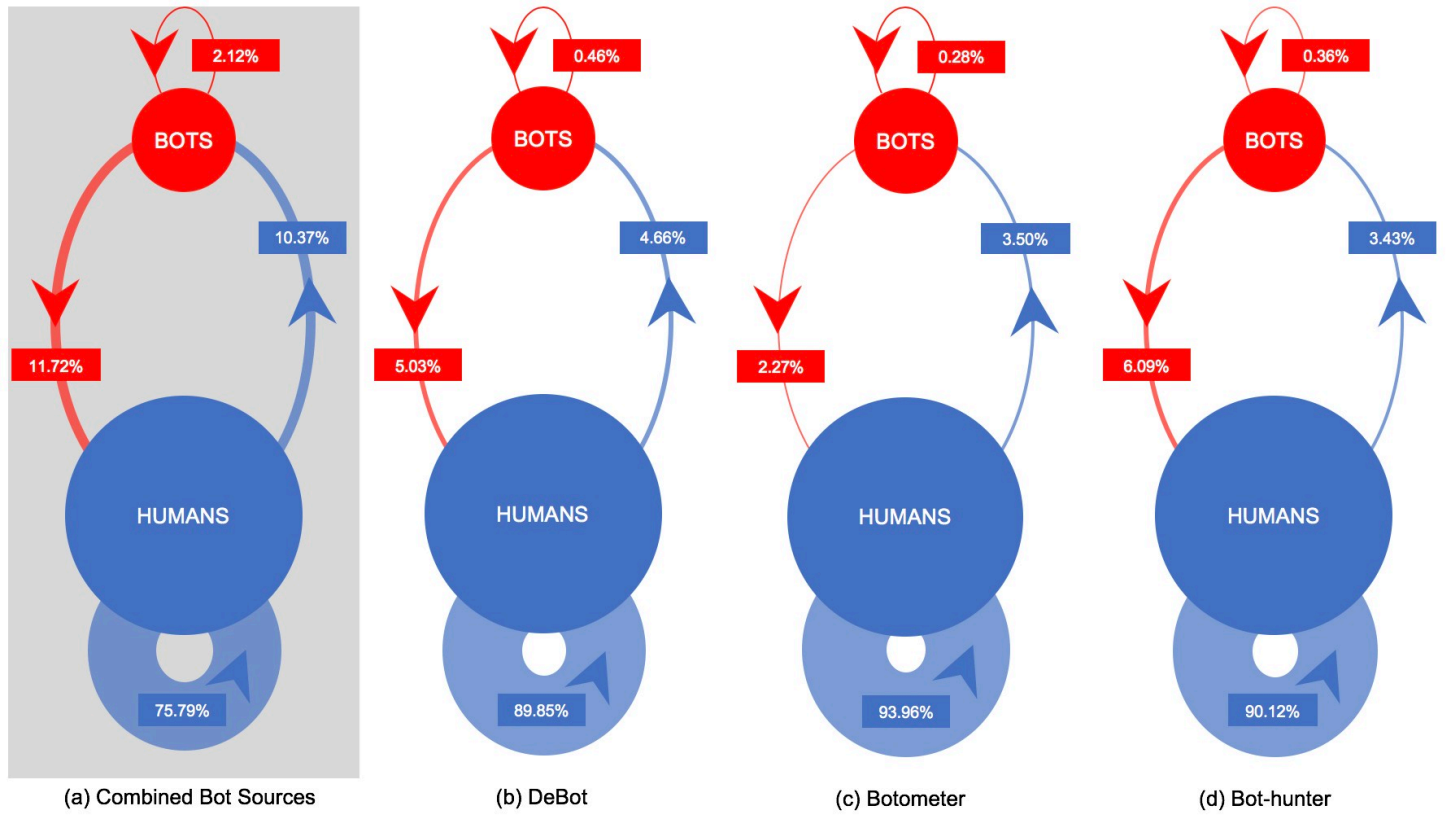
Intra-group and cross-group retweet communication patterns of human (blue) and social bot (red) users within the 2018 U.S. midterm election Twitter conversation according to each bot detection classification platform: (a) Combined Bot Sources (b) DeBot (c) Botometer (d) Bot-hunter. The combined bot sources results (shown in gray) classified an account as a bot in aggregate fashion if any of the three detection platforms classified the account as a bot.

The construction of the election corpus retweet network allowed for the observation of communication interaction patterns between detected bot and human accounts. Fig 4 presents the consolidated intra-group (i.e. bots retweeting bots or humans retweeting humans) and cross-group (i.e. bots retweeting humans or humans retweeting bots) patterns between bot and human accounts from the consolidated aggregate bot perspective, shown in Fig 4A (shaded in gray), as well as individual detection platform perspectives in Fig 4B–4D. Across all bot detection platforms, bot accounts initiate interaction with human accounts at a much higher rate than with other bot accounts, with intra-group bot rates all below 0.50% from the individual detection platform perspective. Social bot accounts detected by DeBot (Fig 4B) and Bot-hunter (Fig 4D) attempt to engage with human accounts at much higher rates than observed with bot accounts detected by Botometer (Fig 4C), thus suggesting the DeBot and Bot-hunter classification algorithms more readily identify bot accounts that are more persistent in engaging in social dialogue with human accounts. While the combined bot sources perspective (Fig 4B) shows that when combining the individual bot detection platform results, minimal overlap or redundancy exists in the consolidated set of detected bots due to the substantially decreased human intra-group rate and increasing rates for all other interactions involving bots. This initial bot coverage assessment is further investigated and discussed in the following Centrality Ranking Coverage subsection.

### Centrality ranking and bot coverage

**Fig 5:**
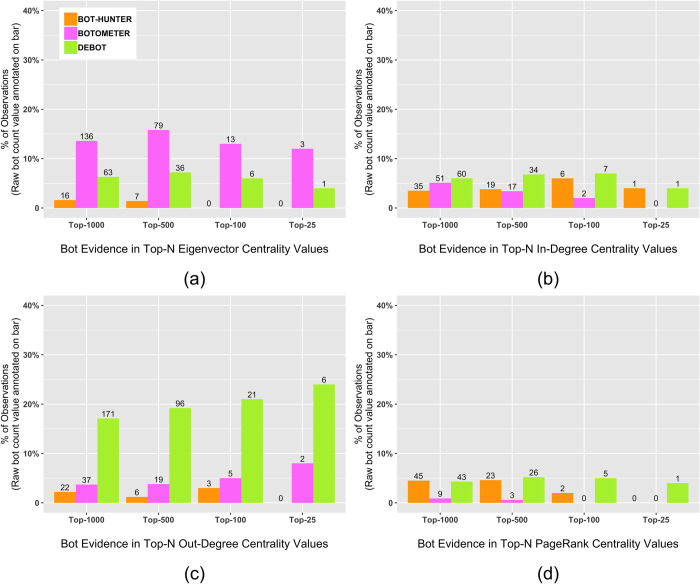
Social bot account evidence within the top-N (where, N = 1000 / 500 / 100 / 25) centrality rankings [(a) eigenvector (b) in-degree (c) out-degree (d) PageRank] according to bot classification results from Bot-hunter (orange), Botometer (pink) and DeBot (green).

Fig 5 presents the centrality ranking analysis results by displaying the density of social bots within the top-*N*, (*where N = 1000 / 500 / 100 / 25*) centrality rankings according to each bot detection platform for the eigenvector, in-degree, out-degree and PageRank centrality measurements. Although social bots detected by DeBot and Botometer accounted for just 0.75% and 3.38% of all unique accounts in the retweet network, respectively, many displayed structural network importance by achieving top centrality out-degree and eigenvector rankings. Specifically, bots detected by DeBot accounted for more than 20% of the top-100 and top-25 out-degree ranking accounts, indicating a persistent social nature for these types of bots. Botometer-detected bots achieved at least 50% more of the top-ranking eigenvector valuations than the other bot detection services. This could imply that Botometer detection techniques discover bots that are highly influential from a structural perspective in a network given their developed direct and indirect relationships with other accounts.

**Fig 6:**
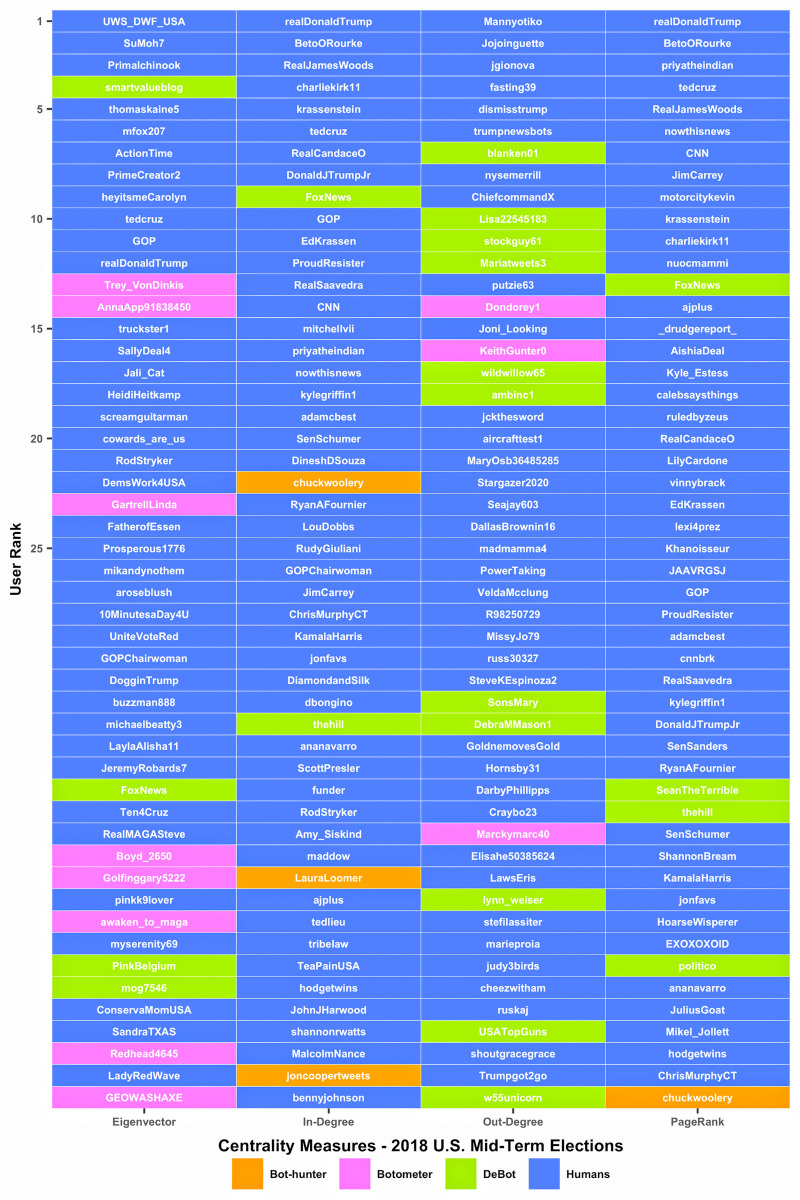
Top-50 bot (orange | pink | green) and human (blue) Twitter accounts within the 2018 U.S. midterm election retweet network ranked by the following four centrality measures: (1) eigenvector, (2) in-degree, (3) out-degree and (4) PageRank.

While all of the bot detection platforms detected few bot accounts within the in-degree and PageRank centrality ranking results, the large variances shown between the out-degree and eigenvector results imply that specific detection methods detect specific types of bots. This concept is further evaluated by directly identifying each bot within the top-50 centrality rankings according to bot detection source and observing potential detection overlap. Fig 6 presents a detection classification ranking visualization with humans colored in blue and suspected bots colored according to their platform detection source. Interestingly, no bots detected within the top-50 rankings for each centrality measurement were detected by more than one detection source. This is further evidence that different detection algorithms are designed to identify different types of bots.

The observation of minimal overlap within the consolidated set of detected bots from the retweet network discussed in the **Intra-group and Cross-group** sub-section, coupled with the lack of detection overlap in the resulting centrality rankings, inspired a final bot coverage assessment of the entire election tweet corpus. The first step of this analysis consisted of a similarity assessment of the bot detection results derived from each of the bot detection platforms used in the study. The Jaccard index (*J*_*A*, *B*_) is a similarity valuation between two sets {A, B} resulting from dividing the intersection of the two sets |*A* ∩ *B*| by their union |*A* ∪ B| as shown in Eq 1.

$$J_{A,B}=\frac{\left|A\cap B\right|}{\left|A\cup B\right|}$$ (1)

**Fig 7:**
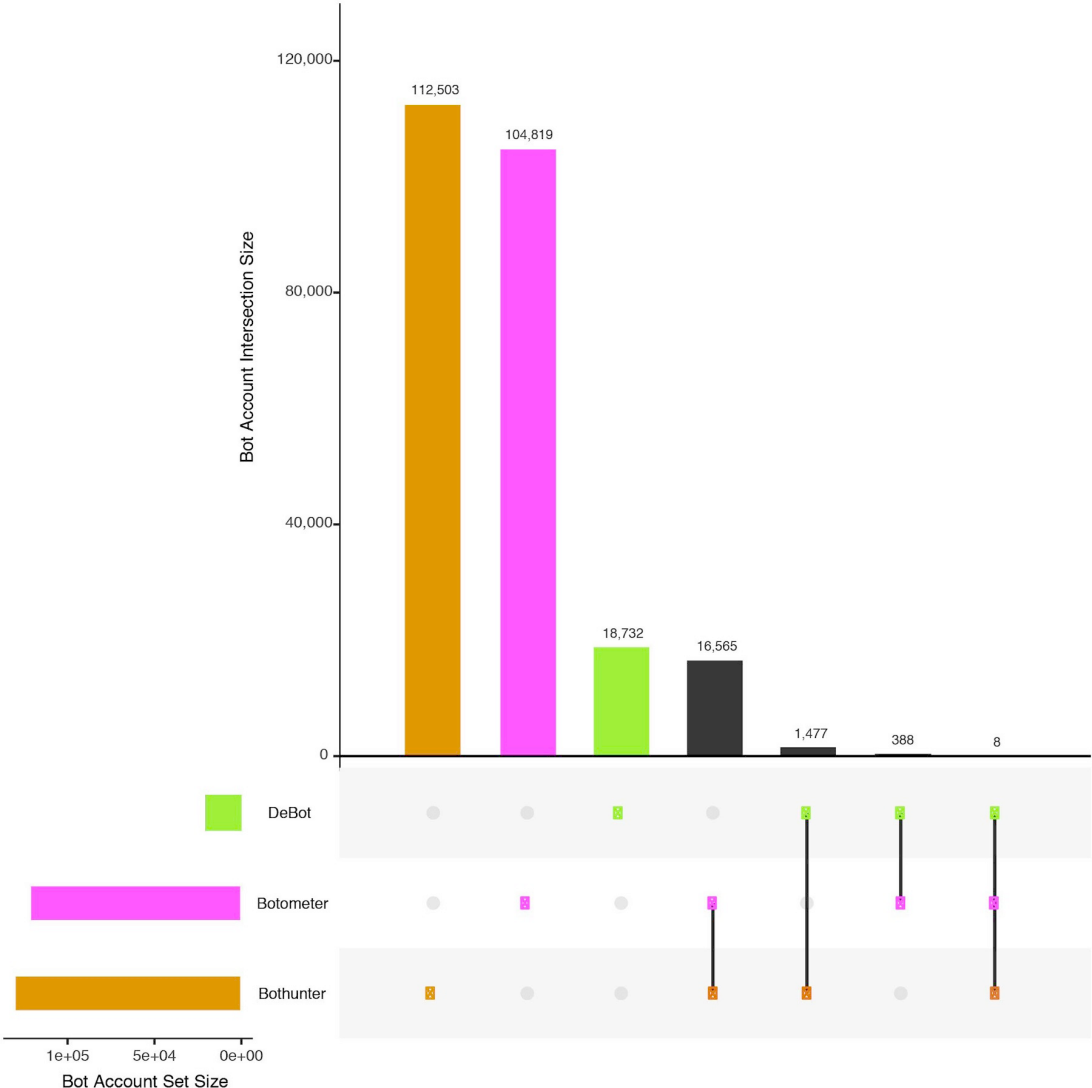
Bot detection coverage analysis for bots detected within the 2018 U.S. midterm election Twitter conversation using the Botometer, Bot-hunter and DeBot bot detection platforms. This figure is based on the UpSet intersection of sets visualization paradigm introduced by Lex et al. [63].

Table 3 presents the Jaccard similarity index results for all possible bot detection platform pairwise comparisons. Overall, there exist minimal levels of overlap between detection platforms as the highest observed similarity value is 7.62% observed between Botometer and Bot-hunter and the similarity values including DeBot are just 0.31% (DeBot and Botometer) and 1.13% (DeBot and Bot-hunter). The UpSet plot show in Fig 7 visually presents the intersection values used to calculate the Jaccard index values, while also identifying a global bot detection overlap of just eight bot accounts between all three bot detection platforms. The top bar chart of the UpSet plot represents the intersection set size between detection results, while the connected dot plots below represent the detection platforms comprising each intersection set volume.

**Table 3:** Jaccard similarity index values representing the pairwise comparison results of the same bots detected between each bot detection platform: Botometer (BT), Bot-hunter (BH) and DeBot (DB).

{*A*, *B*}	|*A* ∩ *B*|	|*A* ∪ *B*|	*J*_*A*, *B*_
DB, BT	388	123,551	0.314%
DB, BH	1,477	131,235	1.125%
BT, BH	16,565	217,322	7.622%

## Conclusion

In summary, this study examined the prevalence and relative importance of detected social bots present within the 2018 U.S. midterm election Twitter conversation. By expanding upon other social bot analysis works, this study incorporated the use of three bot detection platforms in an unprecedented fashion, which enabled a comparative analysis of bot coverage across the Twitter conversation. Bot and human accounts contributed temporally to the 43.5 million tweet election corpus at relatively similar cumulative rates. The intra-group and cross-group analysis of the constructed retweet network showed that bots detected by DeBot and Bot-hunter persistently engaged humans at rates much higher than bots detected by Botometer. Additionally, the intra-group and cross-group interactions, when viewed from a consolidated bot account perspective, provided the first piece of evidence that minimal overall overlap existed between set of bots detected by each detection platform. The centrality ranking results showed that bots, from an overall perspective, achieved many high centrality ranking positions despite their relatively small population size. The classification of relative importance of social bot accounts according to certain centrality results was most notable, with bots detected by DeBot in the out-degree rankings and with bots detected by Botometer in the eigenvector rankings. Analyzing the overlap of bots detected by the detection platforms showed that no overlap existed between the bots ranking in the top-50 centrality results. Moreover, the Jaccard similarity index showed little bot detection overlap from a pairwise perspective, with only eight bots out of a total of 254,492 unique bots in the total tweet corpus having been detected by all three detection platforms.

The overall findings of the study are promising, but not immune from limitations. First of all, the analyzed OSN election corpus relied upon a single platform, Twitter. This reliance surely introduces platform representativeness and sampling bias issues as described in other works [31,32]. Secondly, the keyword categorization of a midterm election is much harder to efficiently account for than to a more specific election like a single congressional or even presidential election. Thus, the keyword filters used to harvest tweets, while attempting to be representative and balanced, surely introduce an unknown level of potential selection bias as detailed by Zhang et al. [55]. Finally, while the focus of the study was on the cross-platform detection of bots via different sources, the ultra-conservative cutoff threshold (i.e. 0.80) focused on high bot precision undoubtedly contributed to an overall lower recall. While acceptable for the scope of this study, future work should seek to extend the cutoff threshold to account for more classification results. Further, bots are not necessarily malicious, as many can be classified as just benign automaton actors; therefore, determining such distinction by relevant platform could be quite beneficial as well.

Future extensions of this work should seek to apply this multi-detection platform framework to other OSN use-cases of interest. This study focused on the most readily available and accessible bot detection platforms, but the rapidly evolving research area of bot detection algorithms can hopefully contribute more accessible detection platforms to the greater research community soon. New options such as these would ideally include emerging detection methods that account for the evolving nature of bots, such as the adversarial approach put forth by Cresci et al. [28]. In addition, detection work must begin accounting for other OSN platforms and expanding beyond Twitter in a similar fashion to the examination of bot evidence in Wikipedia edits conducted by Tsvetkova et al. [64]. Ultimately, this study expands current social bot research by putting forth a reproducible framework to evaluate bots from a multi-detection platform perspective, and the novel analysis methods produce actionable results for analysts to better understand the prevalence and relative importance of detected social bots. Bots play a significant participatory role in online conversations, but significant improvement in bot analysis research remains to understand the implication and effect these automated actors play in influencing human actors. This study plays a crucial role in advancing the body of research dedicated to better understanding the role of social bots in social dialogue.
